# Supplementary material for: Venomix: a simple bioinformatic pipeline for identifying and characterizing toxin gene candidates from transcriptomic data
Source: PeerJ. 2018 Jul 31;6:e5361. doi: 10.7717/peerj.5361 (PMC6074769; doi:10.7717/peerj.5361)
Supplement: Supplemental Information 6 [file peerj-06-5361-s006.gz › FinalOutput_1E-6/Peroxiredoxin-4_1/finaltree.pdf]

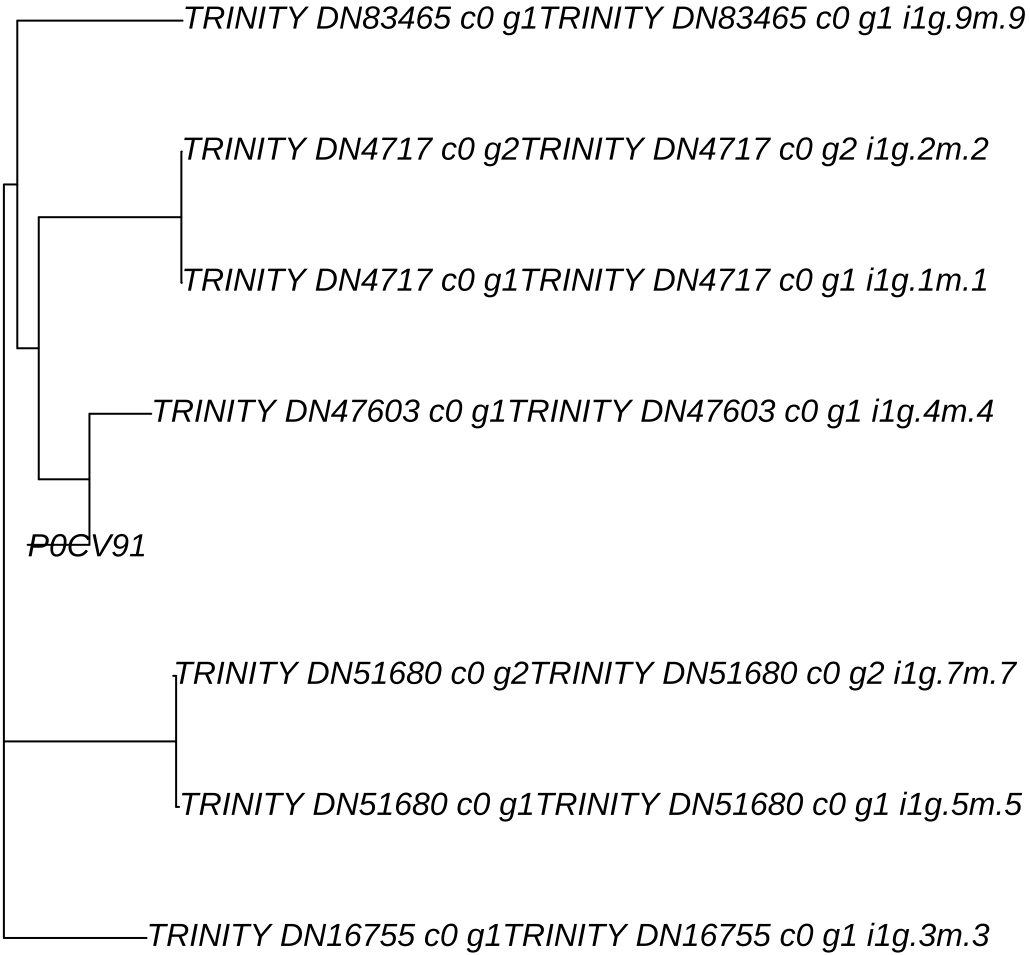
TRINITY DN83465 c0 g1TRINITY DN83465 c0 g1 i1g.9m.9
TRINITY DN4717 c0 g2TRINITY DN4717 c0 g2 i1g.2m.2
TRINITY DN4717 c0 g1TRINITY DN4717 c0 g1 i1g.1m.1
TRINITY DN47603 c0 g1TRINITY DN47603 c0 g1 i1g.4m.4
P0CV91
TRINITY DN51680 c0 g2TRINITY DN51680 c0 g2 i1g.7m.7
TRINITY DN51680 c0 g1TRINITY DN51680 c0 g1 i1g.5m.5
TRINITY DN16755 c0 g1TRINITY DN16755 c0 g1 i1g.3m.3
